# The Interaction of Natural Selection and GC Skew May Drive the Fast Evolution of a Sand Rat Homeobox Gene

**DOI:** 10.1093/molbev/msz080

**Published:** 2019-04-09

**Authors:** Yichen Dai, Peter W H Holland

**Affiliations:** Department of Zoology, University of Oxford, Oxford, United Kingdom

**Keywords:** mutation, biased gene conversion, genome evolution, protein evolution, Pdx1, homeodomain

## Abstract

Several processes can lead to strong GC skew in localized genomic regions. In most cases, GC skew should not affect conserved amino acids because natural selection will purge deleterious alleles. However, in the gerbil subfamily of rodents, several conserved genes have undergone radical alteration in association with strong GC skew. An extreme example concerns the highly conserved homeobox gene *Pdx1*, which is uniquely divergent and GC rich in the sand rat *Psammomys obesus* and close relatives. Here, we investigate the antagonistic interplay between very rare amino acid changes driven by GC skew and the force of natural selection. Using ectopic protein expression in cell culture, pulse-chase labeling, in vitro mutagenesis, and drug treatment, we compare properties of mouse and sand rat Pdx1 proteins. We find that amino acid change driven by GC skew resulted in altered protein stability, with a significantly longer protein half-life for sand rat Pdx1. Using a reversible inhibitor of the 26S proteasome, MG132, we find that sand rat and mouse Pdx1 are both degraded through the ubiquitin proteasome pathway. However, in vitro mutagenesis reveals this pathway operates through different amino acid residues. We propose that GC skew caused loss of a key ubiquitination site, conserved through vertebrate evolution, and that sand rat Pdx1 evolved or fixed a new ubiquitination site to compensate. Our results give molecular insight into the power of natural selection in the face of maladaptive changes driven by strong GC skew.

## Introduction

Vertebrate genomes are far from uniform in their proportions of A, C, G, and T nucleotides and are mosaics of more AT-rich and more GC-rich regions (Eyre-Walker and Hurst 2001). The processes that generate these patterns are complex but include variation in the mutability of certain nucleotides (Ehrlich and Wang 1981), differences in the probabilities of each transition and transversion mutation (Petrov and Hartl 1999; Schaibley et al. 2013), and the influence of chromosome pairing at meiosis through both recombination-associated AT skewed point mutation (Arbeithuber et al. 2015) and by GC-biased gene conversion (gBGC; Galtier 2001; Galtier and Duret 2007; Arbeithuber et al. 2015). The latter is considered to be strong force acting to elevate GC content locally. gBGC is a process in which GC pairs are preferred over AT pairs at polymorphic sites and occurs by biased repair of mismatched DNA heteroduplexes formed by strand invasion after pairing of homologous chromosomes; it leads to gradual accumulation of GC in the affected genomic region (Galtier 2001; Galtier and Duret 2007). gBGC has been shown to be widely present in vertebrates and is a prominent effect leading to local accumulation of GC content, especially in intergenic regions and synonymous third codon positions (Figuet et al. 2015).

The predominant localization of GC skew to synonymous sites (Eyre-Walker and Hurst 2001) implies that natural selection is usually effective at removing GC changes that negatively affect protein function. However, an unusual and extreme case has been discovered recently in gerbil genomes whereby very high GC skew is associated with radical coding sequence divergence in localized regions of the genome (Hargreaves et al. 2017). In these animals, several highly conserved proteins have diverged dramatically over a short evolutionary period through amino acid changes almost always associated with changes from AT- to GC-rich codons. This finding allows us a rare insight into the inevitable conflict between GC skewed nucleotide changes and protein function. For example, some highly conserved sites, inferred to be under strong purifying selection, have undergone unique changes in gerbils. These rare cases offer the chance to explore how molecular evolution might compensate for maladaptive functional changes.

The *pancreatic duodenum homeobox 1* gene (*Pdx1*, also referred to as *Xlox*, *Ipf1*, *Idx1*, *Stf1*, *XlHbox8*, or *Lox*) is a member of the ParaHox gene cluster and one of the most highly conserved of all homeobox genes (Wright et al. 1989; Brooke et al. 1998; Ferrier and Holland 2002). The Pdx1 protein is the key transcription factor initiating vertebrate pancreatic development and is also essential for maintaining pancreatic β-cell identity in adulthood (Jonsson et al. 1994; Ahlgren et al. 1996; Ahlgren et al. 1998). There are two key functional domains in the Pdx1 protein: the hexapeptide domain (PFPWMK) responsible for dimerization with PBC proteins (Liberzon et al. 2004), and the 60 amino acid homeodomain responsible for binding to DNA and sequence-specific recognition of promoter sequences (Lu et al. 1996). Throughout vertebrate evolution, the Pdx1 homeodomain has been highly conserved, retaining 100% amino acid identity between catshark, coelacanth, *Xenopus*, chicken, cat, mouse, and human, and just two to four amino acid changes seen in teleost fish and *Anolis* lizard. This extreme conservation, for over 420 My, suggests that every residue in the 60 amino acid homeodomain is likely to be under strong purifying selection, not just those encoding the three alpha helices key to homeodomain folding.

The importance of the homeodomain sequence is also indicated by human phenotypes. A single amino acid change in the third alpha helix, R197H, is associated with drastically lowered levels of insulin expression and a propensity for heterozygous carriers to develop type II diabetes (Macfarlane et al. 1999). The most extreme phenotype reported is absence of a pancreas in an infant carrying one *Pdx1* allele with a E164D point mutation in the homeodomain and a second allele with the mutation E174K (Schwitzgebel et al. 2003). These changes did not alter protein localization, binding to the *insulin* gene promoter, or interaction with known Pdx1 cofactors, but both mutant proteins had significantly shortened protein half-life as measured in cell culture assays. Given that previous work has shown that Pdx1 is degraded via the ubiquitin 26S-proteasome pathway (Claiborn et al. 2010), it is likely that these mutations altered ubiquitination sites and/or the interaction between Pdx1 and ubiquitin pathway proteins.

The only known exceptions to the extreme conservation of vertebrate *Pdx1* are rodents of the gerbil subfamily. It was recently shown that *Pdx1* is highly divergent in two gerbil species: *Psammomys obesus* (sand rat) and *Meriones unguiculatus* (Mongolian gerbil) (Hargreaves et al. 2017). In these animals, the normally invariant 60 amino acid homeodomain has experienced 15 and 14 amino acid changes, along with drastic change outside the homeodomain. This implies that radical Pdx1 sequence divergence occurred in the ancestry of gerbils. Although amino acid changes would normally be indicative of either reduced selection pressure or positive adaptive change, neither seems likely in this case. Instead, detailed analysis in *Psammomys obesus* reveals that the changes have been driven by extreme accumulation of GC nucleotides across a very extensive genomic region, possibly by gBGC, and that the GC bombardment of the gene may have driven maladaptive changes to fixation. Similar substitutions are not seen in any other animal lineage. This unusual situation gives an opportunity to examine how natural selection responds to intense mutation accumulation. First, we ask whether amino acid changes, driven by GC skew, have affected protein stability, by analogy to some human mutations. Second, we use a pharmacological inhibitor to test if, despite divergence, sand rat protein stability is regulated by the ubiquitin-proteasome system (UPS). Third, we use in vitro mutagenesis to identify which amino acid residues are targeted for ubiquitination and activation of UPS in mouse and sand rat proteins and uncover possible compensatory mutations. Our data suggest that extreme accumulation of GC changes removed a critical and conserved ubiquitination site, and that this maladaptive change was overcome by emergence of a novel compensatory change. Hence, despite an onslaught of GC skew, some critical protein properties are maintained.

## Results

### Altered Stability of Gerbil Pdx1 Protein

**Fig. 1.**
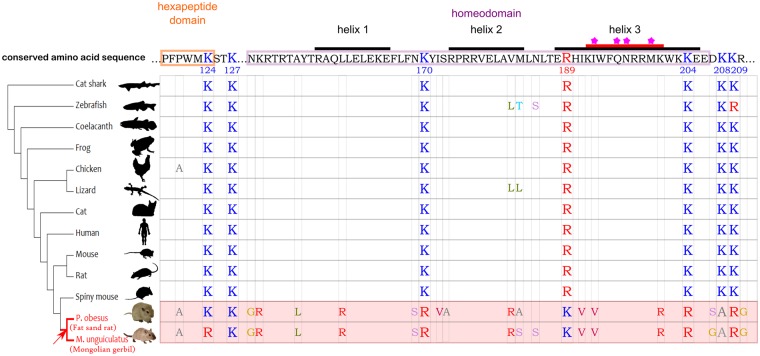
Alignment of Pdx1 hexapeptide and homeodomain sequence across vertebrates. The human and mouse amino acid sequence, conserved in most vertebrates, is shown at the top. The three alpha helices are labeled, and key amino acids known to directly interact with DNA bases are highlighted with a star (Longo et al. 2007). Lysine/Arginine residues key to the current research are shown for all species. For other residues, only those that deviate from the conserved sequence are shown.

The divergence of sand rat and Mongolian gerbil *Pdx1* homeobox sequence, compared with the usual extreme conservation, raises the question of whether the gene is nonfunctional in these species. We have previously shown that this is unlikely since the *Pdx1* coding sequence is depleted in polymorphisms relative to the 3′ untranslated region, suggesting presence of purifying selection (Hargreaves et al. 2017). We also note that five of the six hexapeptide residues are unchanged. As for the homeodomain, the amino acid changes in helix 3, which makes close contact with the major groove of DNA, are between chemically similar amino acids; there is one chemically significant change (Leu to Arg) in helix 1 (fig. 1).This pattern of change suggests that the gene is functional, and that purifying selection has resisted substitutions of residues that affect interactions with DNA. The nonhelix sites that have changed in gerbils are also normally conserved, but in these cases, substitutions driven by GC skew have somehow been tolerated in gerbils only. To test if substitutions are adaptive, we analyzed the hexapeptide and homeodomain regions of the 13 species shown in figure 1, and fitted site, branch, and branch-site models using codeml in PAML (Yang 1997). We found no support for positive selection occurring within the gerbil lineage (Supplementary Material online). A two-ratio branch model gave a d*N*/d*S* ratio of 0.0197 (d*N* = 0.1280, d*S* = 6.0599) on the gerbil lineage, indicating a high rate of synonymous substitution and presence of purifying selection. We suggest the observed amino acid changes in gerbil Pdx1 are maladaptive changes.

**Fig. 2.**
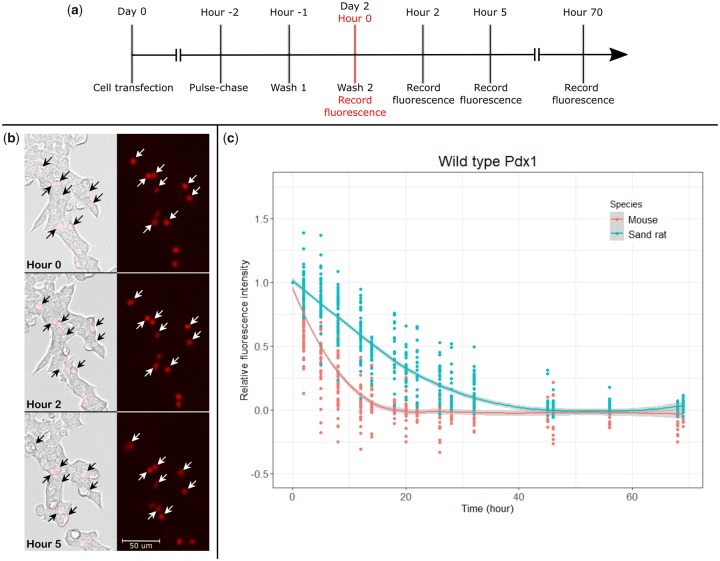
Measurement of protein stability. (*a*) Experimental design showing timing of cell transfection, pulse-chase, and fluorescence recording. (*b*) An example of cell tracking over 5 h. The labeled cells are overexpressing mouse Pdx1-SNAP. (*c*) Intensity decline for mouse Pdx1 and sand rat Pdx1 proteins. Each dot represents an individual cell. The curve shown was generated using the Local Regression (loess) function in the ggplot2 package in R (Wickham 2016).

To assess if Pdx1 protein stability has been altered in gerbil evolution, we conducted pulse-chase labeling experiments of ectopically expressed proteins in a rat pancreatic cell line (INS-1). To allow tracking of protein degradation in individual cells, mouse and sand rat *Pdx1* genes were cloned into pSNAP_f_ vectors, after first lowering the GC content of the sand rat codons (“mousifying”) to optimize translation efficiency (Supplementary Material online). These constructs produce Pdx1 fusion proteins with a C-terminal SNAP-tag motif, which will link covalently to a red fluorescent tag (SNAP-Cell TMR-Star) enabling protein stability to be measured after washing out unbound red tag. After transfection into INS-1 cells, pulse-chase was carried out and fluorescence intensity of individual transfected cells recorded at intervals up to 70 h (fig. 2*a*). Individual cells were identified under brightfield imaging, and cells that exhibited ambiguity in identity were excluded from analysis (fig. 2*b*). Data from 89 (*n* = 43 transfected with mouse *Pdx1*, *n* = 46 transfected with sand rat *Pdx1*) individual cells were used to plot decrease in intensity per cell, revealing a faster rate of intensity decline for mouse Pdx1 than for sand rat Pdx1 protein (fig. 2*c*).

To quantify the difference in protein stability, we modeled the decrease of fluorescence intensity using the Linear and Nonlinear Mixed Effects (nlme) Models package (Pinheiro et al. 2018) in R. This revealed a significant difference between the degradation pattern of mouse Pdx1 and sand rat Pdx1 proteins (*P *=* *0; Supplementary Material online). Based on this model, we estimate the half-life of mouse Pdx1 protein to be 4.7 ± 0.4 h, and the half-life of sand rat Pdx1 protein to be 13.7 ± 0.3 h.

### Sand Rat Pdx1 Protein Degradation Is Regulated by the UPS

**Fig. 3.**
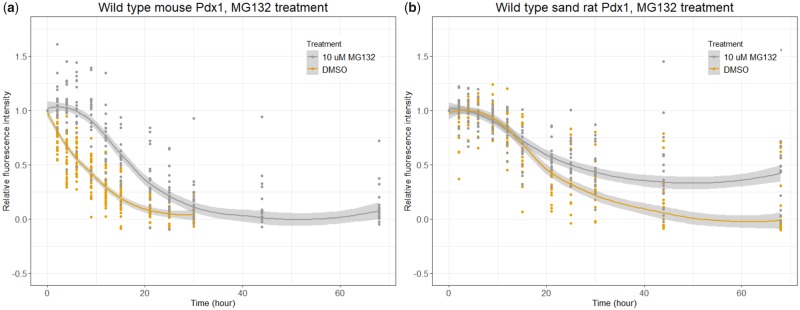
Effect of inhibiting UPS on Pdx1 protein stability. Graphs show the decline in fluorescence intensity for (*a*) mouse Pdx1 and (*b*) sand rat Pdx1 in cells treated with DMSO (control) or 10-µM MG132 (inhibitor of UPS), in both cases showing shift of protein decay curve. Statistical modeling confirmed that in both cases the effect is significant (mouse *P *=* *1e-04; sand rat *P *=* *0.0007); the Pdx1 protein half-lives were extended by 10.8 ± 0.9 h (mouse) and 11.3 ± 1.7 h (sand rat).

It has previously been shown that mouse Pdx1 protein stability is regulated by the UPS (Claiborn et al. 2010). To test if sand rat Pdx1 is regulated by the same biochemical pathway, we treated transfected INS-1 cells with MG132, a reversible inhibitor of the 26S proteasome. MG132 treatment slows the overall rate of mouse Pdx1 protein degradation, shifting the best-fit curve to the right (fig. 3*a*, P* *=* *1e-04; Supplementary Material online). This is consistent with previous findings that mouse Pdx1 protein stability is regulated via UPS. We find that MG132 treatment also slows the rate of sand rat Pdx1 degradation pattern (fig. 3*b*, *P *=* *0.0007; Supplementary Material online), although less sharply compared with mouse Pdx1. Differences in processes prior to proteasome degradation, for example, E3 ligase efficiency in recognizing and tagging the target Pdx1 protein, could contribute to subtle differences in how proteins respond to proteasome inhibition.

### Identification of Pdx1 Ubiquitination Sites Suggests Compensatory Molecular Evolution

Although ubiquitin can sometimes be added to the peptide N-terminus or to cysteine residues, it is only added to lysine residues in the context of targeting for degradation in proteasomes (Kerscher et al. 2006). We therefore asked which lysine residues were utilized by the UPS in regulating stability of mouse and sand rat Pdx1 proteins. We first used prediction software to deduce which residues had the highest probability of being a functional ubiquitination site (Chen et al. 2013; Gao et al. 2013; Wang et al. 2017). In addition, because ubiquitination acts through covalent addition of a moiety to lysine residues, we excluded key lysine residues in the homeodomain that have been proven to engage in interactions with DNA. This resulted in seven lysine residues of interest: four in the conserved Pdx1 protein (mouse, human, and most vertebrates) and three in the divergent sand rat protein.

**Fig. 4.**
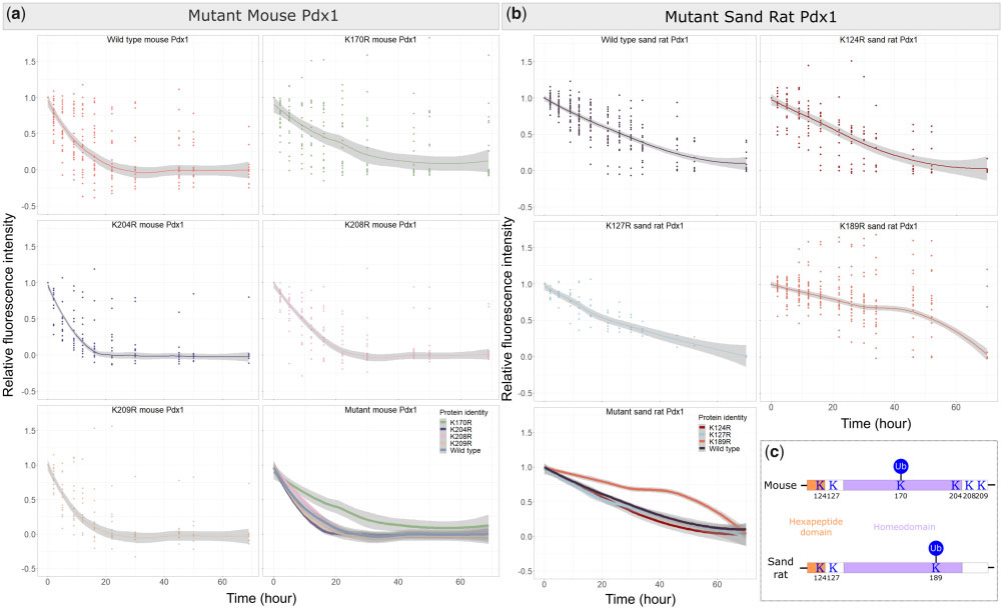
Protein stability comparison between wild type and mutant proteins. (*a*) Graphs show decline in fluorescence intensity for mouse wild type and individual mutant proteins, with a merged graph in the bottom right corner. (*b*) Graphs showing decline in fluorescence intensity for sand rat wild type and individual mutant proteins, with a merged graph in the bottom left corner. (*c*) Summary of ubiquitination site location in mouse and sand rat Pdx1.

We used in vitro mutagenesis to replace singly each lysine codon of interest with a codon for arginine, an amino acid with similar polarity but which cannot be tagged by ubiquitin. For mouse *Pdx1*, we generated mutant proteins K170R, K204R, K208R, and K209R with C-terminal SNAP-tags (numbering according to mouse Pdx1). These constructs were transfected into INS-1 cells and protein stability measured through pulse-chase labeling with SNAP-Cell TMR-Star. As shown in figure 4*a*, mutant proteins K204R, K208R, and K209R did not have significantly different half-lives compared with the wild type control (*P *=* *0.1487, 0.1341, and 0.5847 respectively; Supplementary Material online). However, mutant protein K170R showed a very different protein decay pattern, with a much flatter best-fit decay curve and a longer half-life (*P *=* *0.0267; Supplementary Material online). This reveals that lysine residue 170 is the major Pdx1 ubiquitination site. We suspect it is not the only ubiquitination site as the protein is still gradually degraded within each cell.

For sand rat Pdx1, we generated SNAP-tagged mutant proteins K124R, K127R, and K189R, transfected into INS-1 cells and measured protein stability (numbering according to alignment with the mouse Pdx1 protein). As shown in figure 4*b*, mutant proteins K124R and K127R did not have drastically different stability compared with wild type control (*P *=* *0.9649 and 0.5538, respectively; Supplementary Material online). Only mutant K189R displayed a completely different degradation pattern compared with the other two mutant proteins and the wild type (*P *=* *0.0000; Supplementary Material online). For K189R, high levels of fluorescence were detected in cells throughout the experimental period, with many cells exhibiting rounded shapes and detaching from the substrate around hour 50. This radical disruption of regulated protein decay indicates that the K189 is the ubiquitination site for sand rat Pdx1. Mutation of this site abolishes Pdx1 degradation via the UPS pathway.

**Fig. 5.**
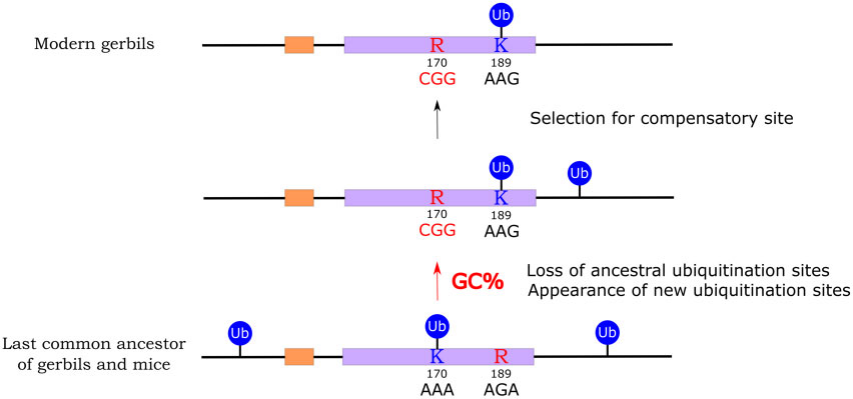
Summary of the possible evolutionary pathway leading to emergence of a compensatory ubiquitination site and loss of conserved sites in gerbil Pdx1. This process is associated with GC skew.

Amino acid 170, which we identify as the major functional ubiquitination site in the mouse Pdx1 protein, has been conserved across 420 My of jawed vertebrate evolution. This K170 site is invariant across vertebrates, with the exception of some teleosts and the gerbil species discussed here. In sand rat and Mongolian gerbil, the codon has undergone a unique, GC skew associated mutation to arginine (fig. 1). In contrast, stability and degradation of sand rat Pdx1 is regulated through ubiquitination of K189, a site which across the rest of jawed vertebrate evolution is otherwise conserved as arginine. These results reveal a unique shift in the location of the ubiquitination site in Pdx1 along the evolutionary lineage leading to gerbils (fig. 4*c*). We propose that in the course of evolution, and under extreme GC skew, the ultra-conserved lysine residue 170, possibly along with other ubiquitination sites, was removed by mutation and as a compensatory mechanism natural selection favored an otherwise maladaptive mutation elsewhere in the protein sequence (fig. 5). The fixation of the “new” lysine 189 is consistent with natural selection acting in the face of strong GC skew.

## Discussion

The sand rat and the Mongolian gerbil are both desert-dwelling rodents, the former native to North Africa and the Middle East (Schmidt-Nielsen et al. 1964) and the latter to Mongolia and China (Mallon 1985). Both are members of the Gerbillinae subfamily that diverged from the Murinae subfamily, including mice and rats, 18 ± 1.8 Ma (Chevret and Dobigny 2005). The sand rat has been of particular interest in studies of diabetes since both wild-caught and laboratory-reared animals can exhibit elevated fasting blood glucose levels, increased body weight and symptoms of type 2 diabetes after being fed a high calorie diet (Schmidt-Nielsen et al. 1964; Shafrir and Gutman 1993). It had been hypothesized that these characteristics are associated with loss of the *Pdx1* gene in the gerbil subfamily (Leibowitz et al. 2001), but our recent work revealed that rather than missing the gene, sand rats and Mongolian gerbils possess a highly divergent *Pdx1* (Hargreaves et al. 2017).

Two features make the sequence divergence of gerbil Pdx1 particularly intriguing. First, the divergence of this normally highly conserved protein is unique, with nothing similar seen in other vertebrates, including arid-adapted rodents such as the closely related spiny mouse *Acomys cahirinus*. Second, the divergence of the gene is clearly associated with a strongly pronounced GC skew that encompasses several genes in a large genomic region (Hargreaves et al. 2017). Our working hypothesis to explain these findings is that focused and extreme gBGC has driven accumulation of GC nucleotides throughout this region, generating maladaptive substitutions. If correct, this hypothesis predicts that natural selection would act to fix compensatory mutations, both within and outside the GC skewed region, to restore biological function.

Before considering the existence of compensatory mutations, we first ask if amino acid changes in gerbil Pdx1 are indeed maladaptive. The evidence from comparative analysis is consistent with this view since large changes to the homeodomain, such as in gerbils, have not been tolerated across the rest of vertebrate evolution or indeed across the Bilateria. In addition, we do not find support for positive selection acting on the hexapeptide region and homeodomain in the gerbil lineage. Direct tests of function, such as gene deletion or experimental manipulation in gerbils, would not answer the question if compensatory changes have occurred elsewhere. Gene replacement in mouse would not give a clear answer for the same reason. Instead, we took a biochemical approach and asked whether protein stability was altered, as reported for some human variants associated with disruption of pancreatic function. Using a cell culture expression system, we found that sand rat Pdx1 does have a different half-life to mouse Pdx1, but surprisingly the change was in the opposite direction from our original expectation. Although two different single amino acid changes in human Pdx1 cause decreased half-life, the radically altered sand rat protein has a longer half-life. Perhaps more importantly, we noted that degradation of sand rat Pdx1 protein follows a steady curve, indicative of actively regulated degradation rather than unregulated decay, and we obtained experimental verification that protein stability is regulated by the UPS. This regulated degradation is another indication that the sand rat *Pdx1* gene encodes a functional protein under selection rather than being a nonfunctional pseudogene. Although these assays were conducted in a heterologous system (a rat cell line), we argue that this is not a confounding factor since the key mediators of Pdx1 degradation, Pdx1 C-terminal inhibiting factor 1 (Pcif1) and the scaffolding protein Cul3 (Claiborn et al. 2010), are highly conserved between rat, mouse, and sand rat (>97% protein similarity between all three species).

To investigate candidate maladaptive mutations, we mapped functional sites responsible for UPS-regulated degradation of the mouse Pdx1 protein. These mutagenesis experiments revealed that one highly conserved lysine, K170, is the critical residue for regulated degradation by UPS. Interestingly, this residue has mutated to an arginine in gerbils and could no longer be used as a site for ubiquitination. Loss of this key ubiquitination site is not seen in any other tetrapod lineage and is likely to have been driven by the gBGC phenomenon described above. However, a new ubiquitinated lysine site has evolved in gerbil Pdx1 proteins, at a different site in the same protein domain, and it is this new site that enables UPS-regulated degradation. We suggest that the maladaptive change to a key amino acid residue, conserved for over 420 My, is compensated for by emergence of a new site fulfilling a similar function. We cannot state that the sand rat Pdx1 protein is as efficient or “optimal” as the conserved or ancestral sequence present before the emergence of the hotpot of GC skew. Indeed, amino acid changes at other residues may affect other biochemical properties. However, we propose that as GC skew gradually altered the sequence of this critical gene during gerbil evolution, natural selection favored substitutions elsewhere in the protein to compensate for loss of a critical biochemical function (fig. 5). This gives us an insight into the power of natural selection even in the face of extreme and unusual nucleotide bias.

## Materials and Methods

### Plasmid Construction and In Vitro Mutagenesis

Full coding sequences for mouse and sand rat *Pdx1* genes were synthesized by GenScript, with alterations in sand rat codon usage to lower GC content (Supplementary Material online). Mouse *Pdx1* was inserted into pSNAP_f_ vector (New England BioLabs) using *Bam*HI and *Xho*I restriction sites, and sand rat *Pdx1* inserted using *Not*I and *Xho*I restriction sites. Stop codons were removed to allow expression of Pdx1-SNAP fusion protein. Successful insertion was verified by Sanger sequencing. In vitro mutagenesis was carried out using polymerase chain reaction.

### Cell Culture and Transfection

INS-1 cells (below 30 passages) were maintained in RPMI-1640 medium, supplemented with 10% fetal bovine serum, 50-µM beta-mercaptoethanol, 1-mM sodium pyruvate, 10-mM HEPES, and 100-U/ml penicillin/streptomycin, at 37 °C/5% CO_2_ and passaged every 4 days. For transfection, cells were seeded into 96-well plates at 60,000 cells/well and transfected 5 h later using Lipofectamine 3000 (ThermoFisher Scientific). For each well, 40-µl DNA-lipid mix containing 400-ng plasmid DNA, 0.8-µl P3000 Reagent, and 1.2-µl Lipofectamine was added and incubated overnight.

### SNAP-Staining and Fluorescence Recording

SNAP-Cell TMR-Star dye (New England BioLabs) was diluted 1:400 in culture medium. Fifty microliters was added to each well and incubated at room temperature for 5 min before three washes. Stained cells were incubated in fresh culture medium for 1 h at 37 °C, twice, to ensure dissociation of unbound SNAP-Cell TMR-Star dye prior to analysis. At each timepoint, pictures were taken using a ZOE Fluorescent Cell Imager (Bio-Rad) with fixed settings: brightfield gain = 18, exposure = 300, LED = 40, contrast = 0; red channel gain = 40, exposure = 500, LED = 50, contrast = 0. Image analysis was performed using ImageJ (Schneider et al. 2012).

### MG132 Treatment

Ten-millimolar MG132 stock solution in dimethyl sulfoxide (DMSO, Sigma-Aldrich) was diluted to 10-µM in treatment wells at hour −2. Culture medium containing 10-µM MG132 was used for all medium changes until hour 4, giving a total of 6-h treatment. Controls were treated in parallel using DMSO.

### Statistical Analysis

We used nlme 3.1-137 (Pinheiro et al. 2018) in R 3.5.1 (R Core Team 2018) to model the pattern of protein degradation based on data collected for all protein and treatment types. Square-root transformed data were used to ensure no obvious deviations from homoscedasticity or normality (Supplementary Material online). The linear mixed effects model was constructed using the interaction between sqrt(Time) and type of species as the fixed effect, and the random effect was cell identity. Different experiments were analyzed individually using the same fixed and random effects. Parameters reported are given in Supplementary Material online. Half-life predictions were made for each individual cell using the model generated and the generic predict function in R (R Core Team 2018); half-lives of mouse and sand rat Pdx1 proteins are reported as mean ± standard deviation.

## Supplementary Material

Supplementary data are available at *Molecular Biology and Evolution* online.

## Supplementary Material

Supplement_Material_msz080
