# Supplementary material for: The Interaction of Natural Selection and GC Skew May Drive the Fast Evolution of a Sand Rat Homeobox Gene
Source: Mol Biol Evol. 2019 Apr 9;36(7):1473–80. doi: 10.1093/molbev/msz080 (PMC6573468; doi:10.1093/molbev/msz080)
Supplement: Supplement_Material_msz080 [file supplement_material_msz080.pdf]

Supplementary Material for:

The interaction of natural selection and GC skew may drive

the fast evolution of a sand rat homeobox gene

Yichen Dai and Peter W H Holland

Content:

Section 1: Coding sequences of mouse *Pdx1*, sand rat *Pdx1* and mousified sand rat *Pdx1*

Section 2: PAML analysis for positive selection in Pdx1 hexapeptide and homeodomain

Section 3: Statistical analysis for comparison of mouse and sand rat Pdx1 protein stability

Section 4: Statistical analysis for assessing effect of inhibiting UPS on mouse Pdx1 stability

Section 5: Statistical analysis for assessing effect of inhibiting UPS on sand rat Pdx1 stability

Section 6: Statistical analysis for assessing effect of lysine mutagenesis on mouse Pdx1 stability

Section 7: Statistical analysis for assessing effect of lysine mutagenesis on sand rat Pdx1 stability

Section 1: Coding sequences of mouse *Pdx1*, sand rat *Pdx1* and ‘mousified’ sand rat *Pdx1*

>Mouse *Pdx1*, original sequence (63.4% GC) used for experiments. NCBI Accession NM_008814.3

```
ATGAACAGTGAGGAGCAGTACTACGCGGCCACACAGCTCTACAAGGACCCGTGCGCATTCCAGAGGGG
CCCGGTGCCAGAGTTCAGCGCTAACCCCCCTGCGTGCCTGTACATGGGCCGCCAGCCCCCACCTCCGC
CGCCACCCCAGTTTACAAGCTCGCTGGGATCACTGGAGCAGGGAAGTCCTCCGGACATCTCCCCATAC
GAAGTGCCCCCGCTCGCCTCCGACGACCCGGCTGGCGCTCACCTCCACCACCACCTTCCAGCTCAGCT
CGGGCTCGCCCATCCACCTCCCGGACCTTTCCCGAATGGAACCGAGCCTGGGGGCCTGGAAGAGCCCA
ACCGCGTCCAGCTCCCTTTCCCGTGGATGAAATCCACCAAAGCTCACGCGTGGAAAGGCCAGTGGGCA
GGAGGTGCTTACACAGCGGAACCCGAGGAAAACAAGAGGACCCGTACTGCCTACACCCGGGCGCAGCT
GCTGGAGCTGGAGAAGGAATTCTTATTTAACAAATACATCTCCCGGCCCCGCCGGGTGGAGCTGGCAG
TGATGTTGAACTTGACCGAGAGACACATCAAAATCTGGTTCCAAAACCGTCGCATGAAGTGGAAAAAA
GAGGAAGATAAGAAACGTAGTAGCGGGACCCCGAGTGGGGGCGGTGGGGGCGAAGAGCCGGAGCAAGA
TTGTGCGGTGACCTCGGGCGAGGAGCTGCTGGCAGTGCCACCGCTGCCACCTCCCGGAGGTGCCGTGC
CCCCAGGCGTCCCAGCTGCAGTCCGGGAGGGCCTACTGCCTTCGGGCCTTAGCGTGTCGCCACAGCCC
TCCAGCATCGCGCCACTGCGACCGCAGGAACCCCGGTGA
```

>Sand rat *Pdx1*, original sequence (72.9% GC)

```
ATGGACAGAGAGGCCGAGCCCTTCTTCGAGGCCTCCTGGGCGTTCCCGGGGCCCGAGTTCGCGGCCCC
CGCCTCCTCCTGCCTGTTCGAGGGTGGGGGCGGGCAGCCTCCCCCCCACGCTCCTCCCCACGCTCCTC
CCCACCTCGCCCCGTGCTCCCTGGACCCCACCGGCCTCCAGCCGCCCCAGCCCGGGGTCCCCCCGCCG
CCACCCGGGGGCCCCGACCAACCGCCCTTTGCCTGGATGAAGAGCAGCAAAGGCCAAGCCTGGAGCGG
CCAGTGGGCAGCCCCCGGCCGCAGGACTCGAACCCTGTACACGCGGGCGCAGCGGCTGGAGCTGGAGA
AGGAATTCCTCTTCAGCCGCTACGTCGCGCGGCCGCGGCGCGTGGAGCTCGCGCGGGCGCTGAACCTC
ACCGAGAAGCACGTGAAGGTCTGGTTCCAGAACCGCCGCATGCGCTGGAAGAGGGAGGAGTCCGCGCG
GGGGAGGACGGCGCCCCGGGAGGACGGAGGAGCGGGAGGCTCCCCGCCACCGTCCTCCTCCTCCT
CCTCCTCCTCCGTGGCCCGGGATGCTCCTCCTCTTCTTCTCCCTCCTCCTCCTCCTCCTACGGGGGGA
CTGCGGTGA
```

>Sand rat *Pdx1*, ‘mousified’ sequence (63.0% GC) used for experiments in this study

```
ATGGACAGAGAAGCTGAACCTTTCTTCGAAGCAAGTTGGGCATTCCCTGGTCCAGAGTTCGCGGCTCC
CGCCTCCAGTTGCCTGTTCGAGGGTGGAGGTGGACAGCCTCCACCTCACGCTCCTCCACACGCTCCTC
CACACCTCGCCCCATGCTCCCTGGACCCAACCGGCCTCCAGCCACCTCAGCCAGGAGTCCCTCCGCCA
CCACCTGGAGGCCCTGACCAACCTCCTTTTGCCTGGATGAAAAGCAGCAAAGGCCAAGCCTGGAGCGG
CCAGTGGGCAGCCCCTGGCCGCAGGACTCGAACACTGTACACTCGGGCGCAGCGGCTGGAGCTGGAGA
AGGAATTCTTATTTAGCCGCTACGTCGCGCGGCCTCGGCGCGTGGAGCTCGCACGGGCGTTGAACTTG
ACCGAGAAGCACGTGAAAGTCTGGTTCCAAAACCGTCGCATGCGCTGGAAAAGGGAGGAATCCGCGCG
GGGAAGGACGGCGCCTCGGGAAGACGGAGGAGCGGGAGGTTCTCCTCCACCTAGTAGTTCAAGCTCTA
GTTCCAGCTCCGTGGCCCGGGATGCTCCTCCACTTCTTCTCCCTCCACCACCTCCGCCAACGGGTGGA
CTGCGATGA
```

Section 2: PAML analysis for positive selection in Pdx1 hexapeptide and homeodomain

The alignment and phylogenetic tree in Fig. 1 (main paper) was used for analysis using codeml in PAML (version 4.9) (Yang 1997). First, we tested for positive selection on sites in the hexapeptide region and homeodomain by comparing a null model (M0, one ω for all sites) to a nearly neutral model (M1a, two classes of sites $0 < \omega < 1$ and $\omega = 1$), and comparing M1a to positive selection (M2a, three classes of sites $0 < \omega < 1$, $\omega = 1$, $\omega > 1$). We also compared the beta model (M7, multiple categories $0 < \omega < 1$ and $\omega = 1$) to beta with positive selection (M8, multiple categories $0 < \omega < 1$, $\omega = 1$, plus $\omega > 1$). Models M2a and M8 did not significantly outperform models M1a and M7. Second, we tested for presence of positive selection on the gerbil lineage. We compared the null model (M0) to a two-ratio branch model (gerbil lineage as foreground); the latter did not significantly outperform the null. Third, we used branch-site models to test for positive selection on the gerbil lineage at different sites within Pdx1. We compared a branch-site null model (A1, allowing $0 < \omega < 1$, $\omega = 1$) to a branch-site alternative model (A, allowing $0 < \omega < 1$, $\omega = 1$, and $\omega > 1$); the latter did not significantly outperform the null model. Significance was calculated using the Likelihood Ratio Test (LRT) (Jeffares et al. 2015).

**Model**	**Parameters specified**	**lnL**	**ntime**	**np**
**Site models**				
M0 (one-ratio)	NSsites = 0, model = 0	-1034.558186	23	25
M1a (nearly neutral)	NSsites = 1, model = 0	-1034.558846	23	26
M2a (positive selection)	NSsites = 2, model = 0	-1037.646198	23	28
M7 (beta)	NSsites = 7, model = 0	-1025.473872	23	26
M8 (beta and ω)	NSsites = 8, model = 0	-1025.474532	23	28
**Branch models**				
Two-ratio model	NSsites = 0, model = 2	-1034.061948	23	26
**Branch-site models**				
Model A1 (null model)	NSsites = 2, model = 2, fixomega = 1, omega = 1	-1031.077597	23	27
Model A (alternative model)	NSsites = 2, model = 2, fixomega = 0,	-1031.077597	23	28

Supplementary Table 1. Parameters reported for PAML analysis (version 4.9) (Yang 1997) generated using sequence alignments of Pdx1 hexapeptide region and homeodomain from the 13 vertebrate species shown in Figure 1. The tree used for analysis is shown in Figure 1. The analysis showed that models allowing positive selection did not significantly outperform models not allowing positive selection.

Section 3: Statistical analysis for comparison of mouse and sand rat Pdx1 protein stability

**Fixed effects**	**df**	***t* Value**	***p* Value**
**Intercept**	625	38.32785	0
**Type: Sand rat**	87	12.22414	0
**Sqrt(Time)**	625	-23.24835	0
**Type: Sand rat × Sqrt(Time)**	625	-5.24269	0

Supplementary Table 2. Parameters reported for the linear mixed effects model generated using data shown in Fig. 2c. Table shows degrees of freedom, *t* value, and *p* value of fixed effects sqrt(Time), type of species, and their interaction. Half-life calculations were obtained using this model.

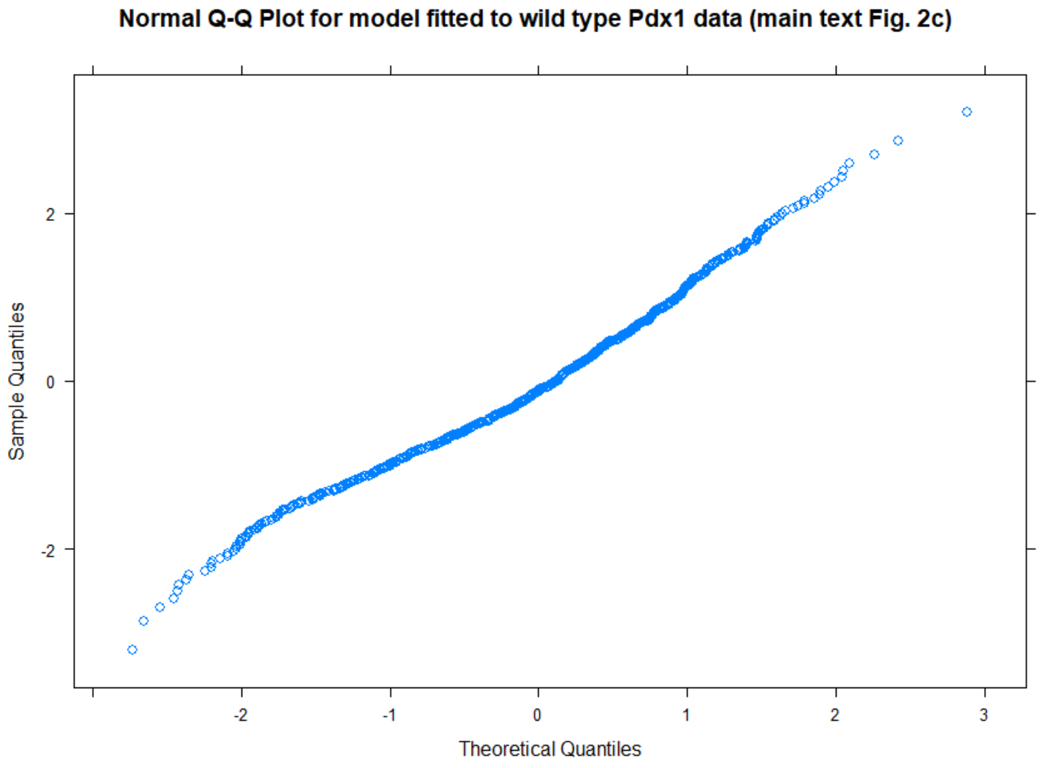

Supplementary Figure 1. To analyse the normality of the square-root transformed data, Quantile-Quantile Plots were generated using the generic qqnorm function in R (R Core Team 2018). X-axis values represent a theoretical group of values following a normal distribution, and Y-axis values represent the values used to construct the linear mixed effects model.

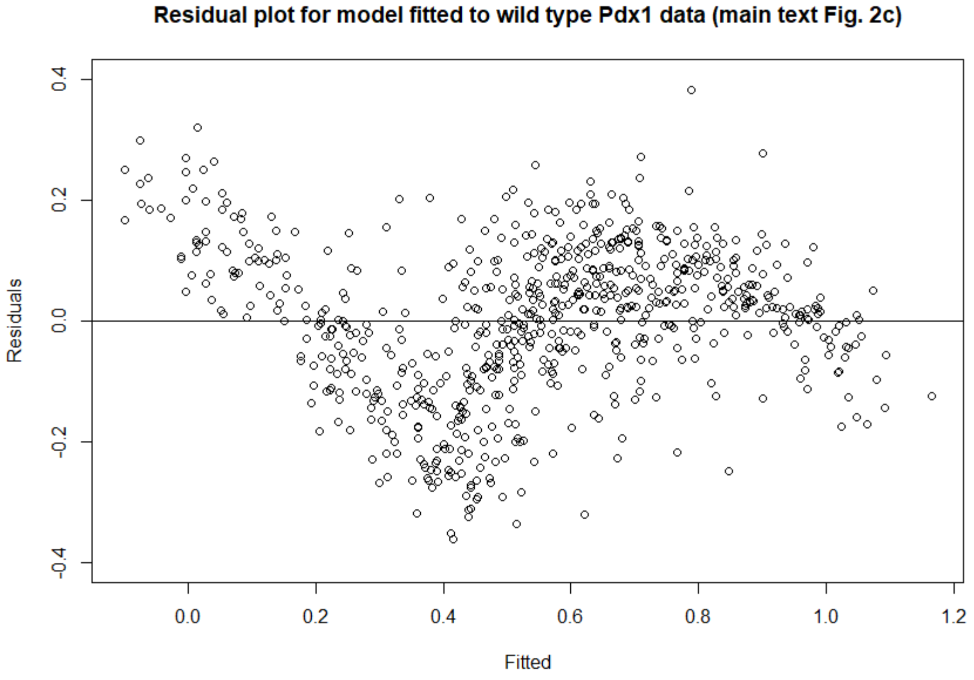

Supplementary Figure 2 To analyse the homoscedasticity of the square-root transformed data, residual plots were generated using the generic plot function in R (R Core Team 2018). The fitted values generated from each linear mixed effects model were plotted back against the difference between each fitted value and original input value.

Section 4: Statistical analysis for assessing effect of inhibiting UPS on mouse Pdx1 stability

**Fixed effects**	**df**	***t* Value**	***p* Value**
**Intercept**	350	38.85098	0e+00
**Type: Treatment**	44	3.67540	6e-04
**Sqrt(Time)**	350	-22.66775	0e+00
**Type: Treatment × Sqrt(Time)**	350	3.96114	1e-04

Supplementary Table 3. Parameters reported for the linear mixed effects model generated using data shown in Fig. 3a. Table shows degrees of freedom, *t* value, and *p* value of fixed effects sqrt(Time), type of treatment, and their interaction. Half-life calculations were obtained using this model.

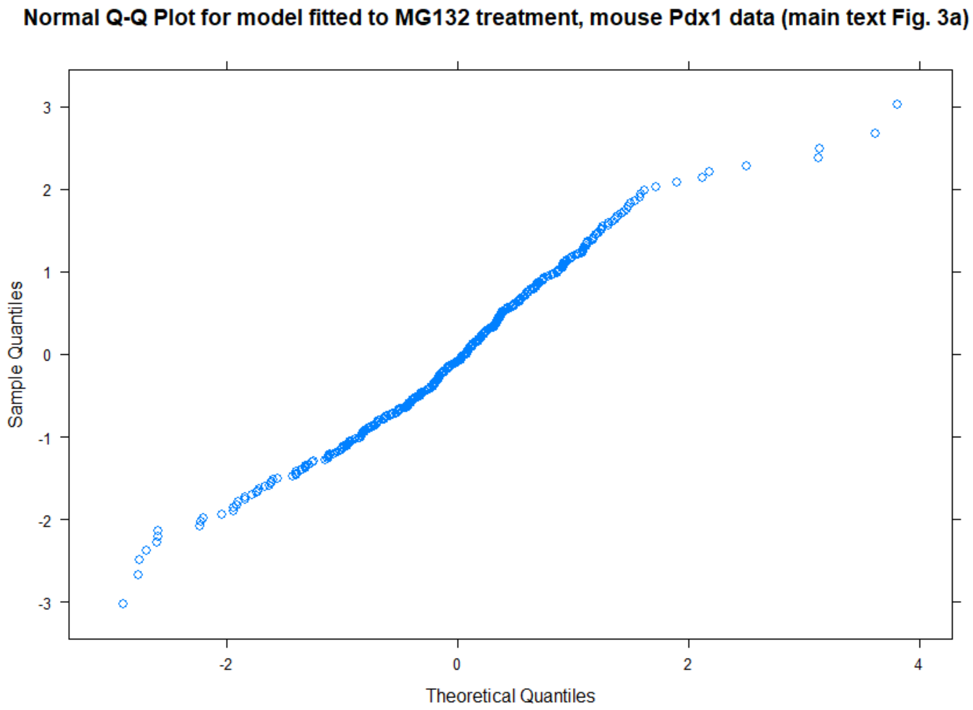

Supplementary Figure 3. To analyse the normality of the square-root transformed data, Quantile-Quantile Plots were generated using the generic qqnorm function in R (R Core Team 2018). Axes as in Supplementary Figure 1.

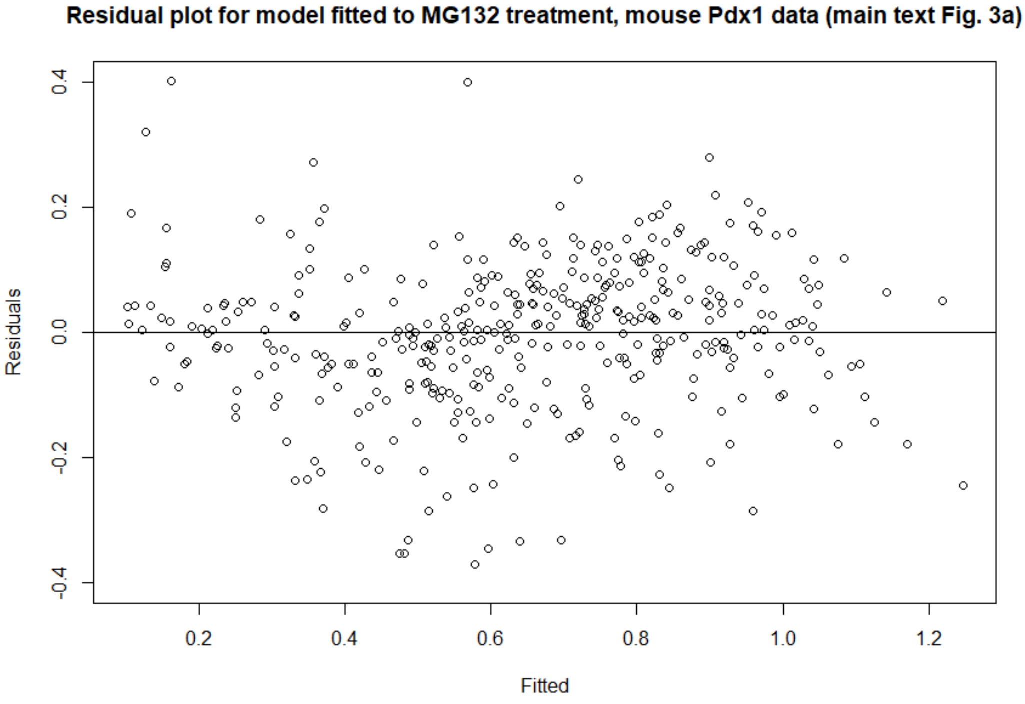

Supplementary Figure 4. To analyse the homoscedasticity of the square-root transformed data, residual plots were generated using the generic plot function in R (R Core Team 2018).

Section 5: Statistical analysis for assessing effect of inhibiting UPS on sand rat Pdx1 stability

**Fixed effects**	**df**	***t* Value**	***p* Value**
**Intercept**	325	36.56956	0.0000
**Type: Treatment**	33	-0.98462	0.3320
**Sqrt(Time)**	325	-16.39190	0.0000
**Type: Treatment × Sqrt(Time)**	325	3.40934	0.0007

Supplementary Table 4. Parameters reported for the linear mixed effects model generated using data shown in Fig. 3b. Table shows degrees of freedom, *t* value, and *p* value of fixed effects sqrt(Time), type of treatment, and their interaction. Half-life calculations were obtained using this model.

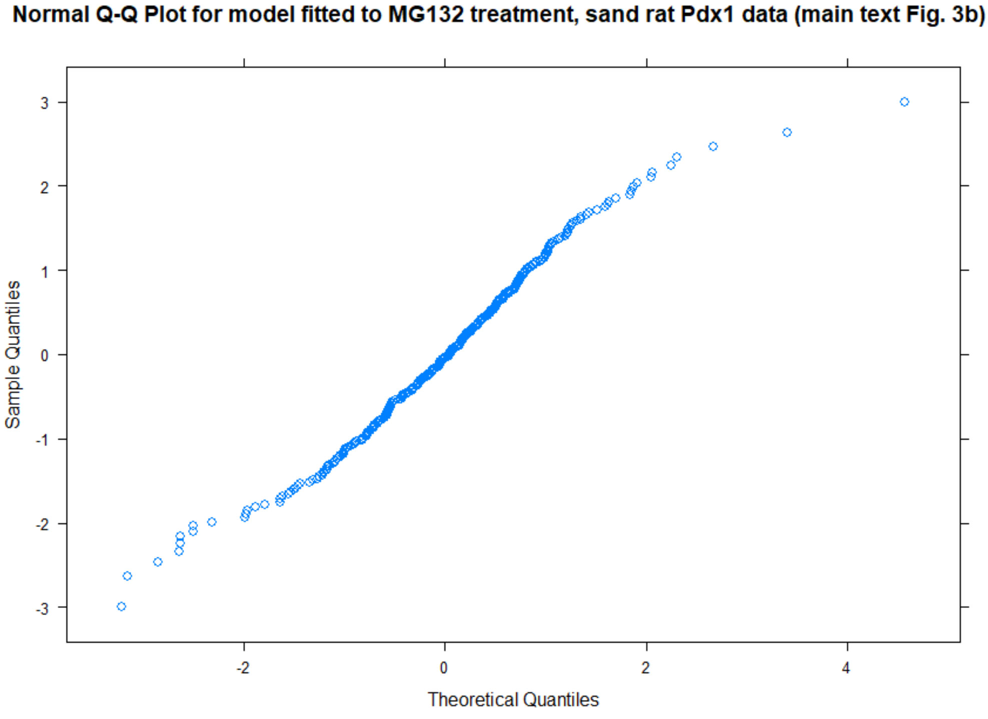

Supplementary Figure 5. To analyse the normality of the square-root transformed data, Quantile-Quantile Plots were generated using the generic qqnorm function in R (R Core Team 2018). Axes as in Supplementary Figure 1.

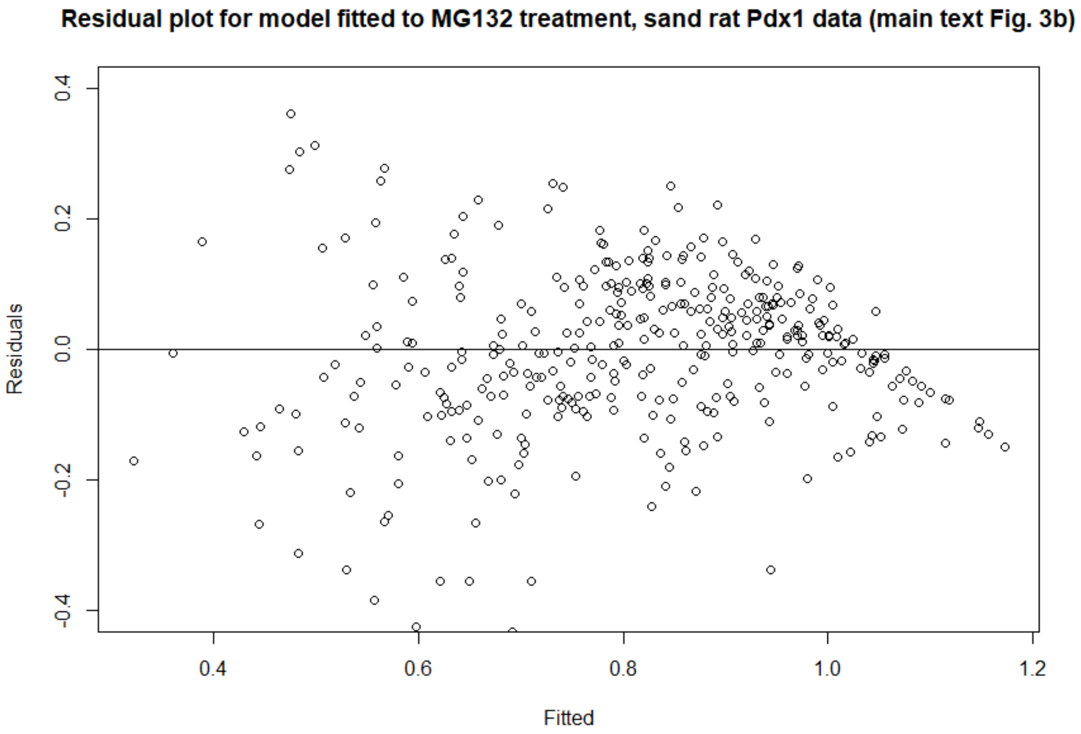

Supplementary Figure 6. To analyse the homoscedasticity of the square-root transformed data, residual plots were generated using the generic plot function in R (R Core Team 2018).

Section 6: Statistical analysis for assessing effect of lysine mutagenesis on mouse Pdx1 stability

Fixed effects	df	t Value	p value
**Intercept**	480	18.953975	0.0000
**Type: K170R**	89	-0.153405	0.8784
**Type: K204R**	89	-0.444863	0.6575
**Type: K208R**	89	1.037924	0.3021
**Type: K209R**	89	0.336021	0.7376
**Sqrt(Time)**	480	-9.084160	0.0000
**Type: K170R × Sqrt(Time)**	480	2.223133	0.0267
**Type: K204R × Sqrt(Time)**	480	-1.446534	0.1487
**Type: K208R × Sqrt(Time)**	480	-1.500572	0.1341
**Type: K209R × Sqrt(Time)**	480	-0.546916	0.5847

Supplementary Table 5. Parameters reported for the linear mixed effects model generated using data shown in Fig. 4a. Table shows degrees of freedom, *t* value, and *p* value of fixed effects sqrt(Time), type of treatment, and their interaction. Half-life calculations were obtained using this model.

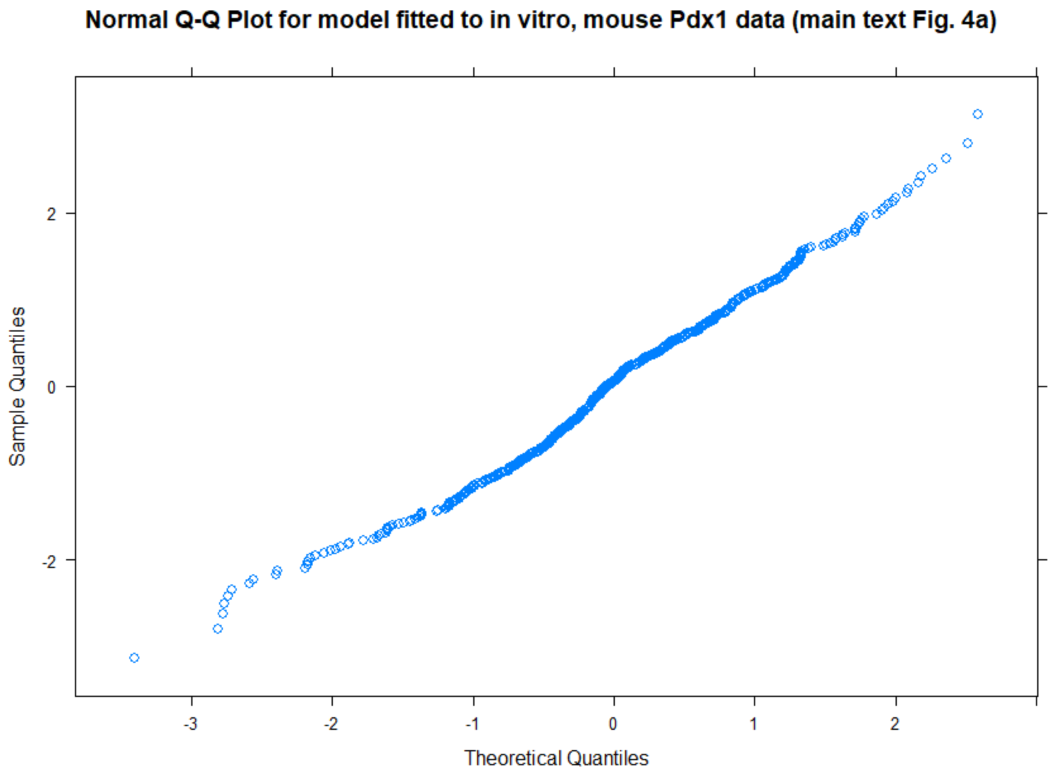

Supplementary Figure 7. To analyse the normality of the square-root transformed data, Quantile-Quantile Plots were generated using the generic qqnorm function in R (R Core Team 2018). Axes as in Supplementary Figure 1.

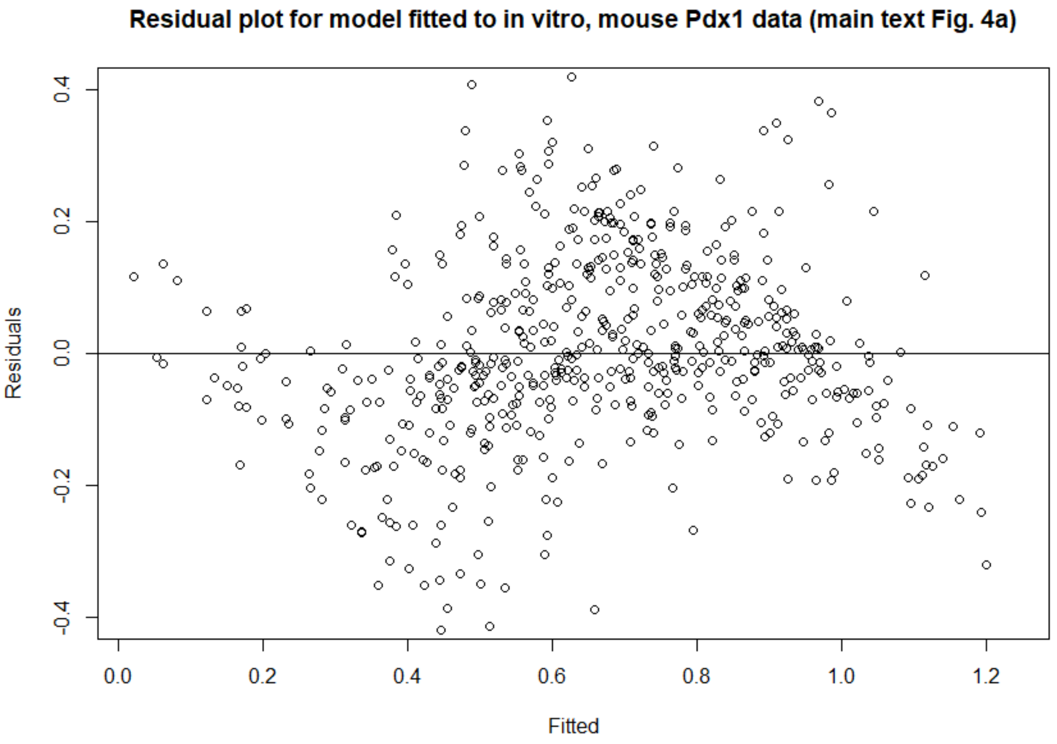

Supplementary Figure 8. To analyse the homoscedasticity of the square-root transformed data, residual plots were generated using the generic plot function in R (R Core Team 2018).

Section 7: Statistical analysis for assessing effect of lysine mutagenesis on sand rat Pdx1 stability

Source of variation	df	*t* Value	*p* value
**Intercept**	626	45.08517	0.0000
**Type: K124R**	84	-1.01502	0.3130
**Type: K127R**	84	-0.67135	0.5038
**Type: K189R**	84	-3.28740	0.0015
**Sqrt(Time)**	626	-21.83059	0.0000
**Type: K124R × Sqrt(Time)**	626	0.04408	0.9649
**Type: K127R × Sqrt(Time)**	626	0.59238	0.5538
**Type: K189R × Sqrt(Time)**	626	11.01595	0.0000

Supplementary Table 6. Parameters reported for the linear mixed effects model generated using data shown in Fig. 4b. Table shows degrees of freedom, *t* value, and *p* value of fixed effects sqrt(Time), type of treatment, and their interaction. Half-life calculations were obtained using this model.

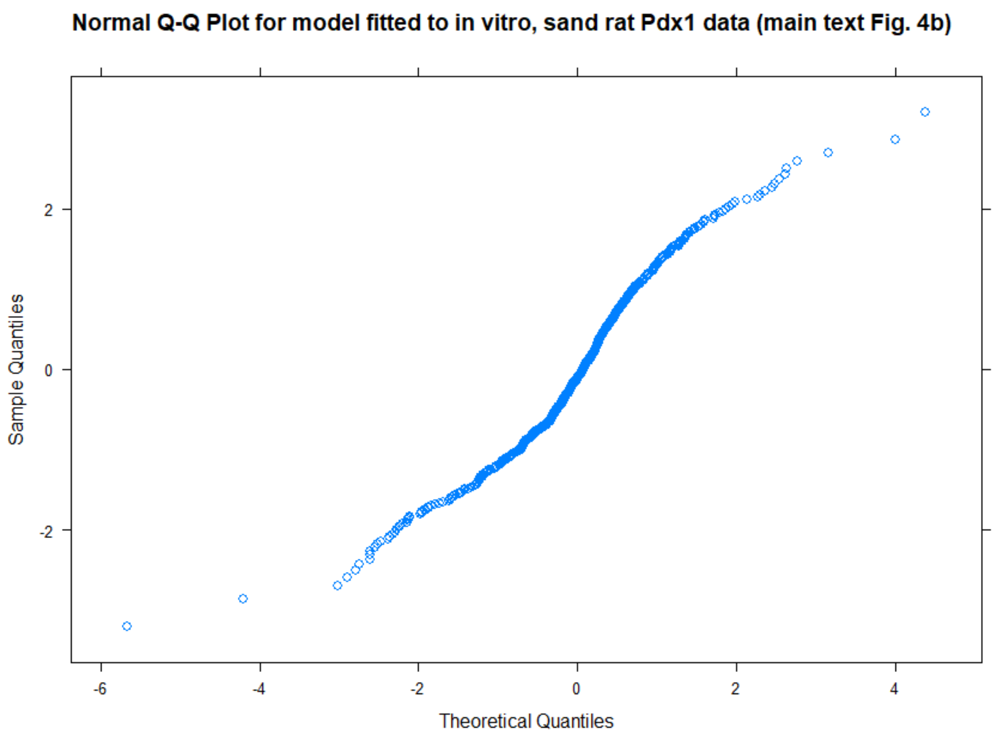

Supplementary Figure 9. To analyse the normality of the square-root transformed data, Quantile-Quantile Plots were generated using the generic qqnorm function in R (R Core Team 2018). Axes as in Supplementary Figure 1.

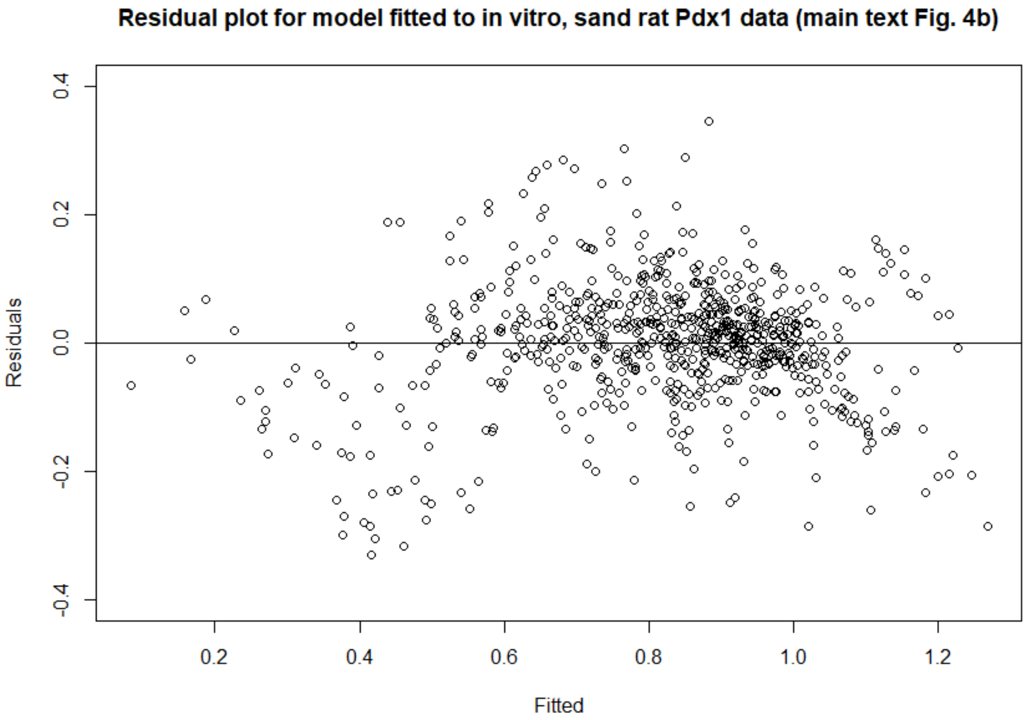

Supplementary Figure 10. To analyse the homoscedasticity of the square-root transformed data, residual plots were generated using the generic plot function in R (R Core Team 2018).

References

Jeffares DC, Tomiczek B, Sojo V, dos Reis M. 2015. A Beginners Guide to Estimating the Non-synonymous to Synonymous Rate Ratio of all Protein-Coding Genes in a Genome. In: Peacock C, editor. Parasite Genomics Protocols. New York, NY: Springer New York. p. 65–90.

R Core Team. 2018. R: A Language and Environment for Statistical Computing.

Yang Z. 1997. PAML: a program package for phylogenetic analysis by maximum likelihood. Comput Appl Biosci. 13(5):555–6. doi:10.1093/bioinformatics/13.5.555.
